# The Evidence Project risk of bias tool: assessing study rigor for both randomized and non-randomized intervention studies

**DOI:** 10.1186/s13643-018-0925-0

**Published:** 2019-01-03

**Authors:** Caitlin E. Kennedy, Virginia A. Fonner, Kevin A. Armstrong, Julie A. Denison, Ping Teresa Yeh, Kevin R. O’Reilly, Michael D. Sweat

**Affiliations:** 1 Social and Behavioral Interventions Program, Department of International Health, Johns Hopkins Bloomberg School of Public Health, 615 North Wolfe Street, Room E5547, Baltimore, MD 21205 USA; 2 Division of Global and Community Health, Department of Psychiatry and Behavioral Sciences, Medical University of South Carolina, 176 Croghan Spur Road, Suite 104, Charleston, SC 29407 USA

**Keywords:** Risk of bias, Study quality, Study rigor, Quality assessment, Rigor assessment, Rigor score, Critical appraisal

## Abstract

**Background:**

Different tools exist for assessing risk of bias of intervention studies for systematic reviews. We present a tool for assessing risk of bias across both randomized and non-randomized study designs. The tool was developed by the Evidence Project, which conducts systematic reviews and meta-analyses of behavioral interventions for HIV in low- and middle-income countries.

**Methods:**

We present the eight items of the tool and describe considerations for each and for the tool as a whole. We then evaluate reliability of the tool by presenting inter-rater reliability for 125 selected studies from seven published reviews, calculating a kappa for each individual item and a weighted kappa for the total count of items.

**Results:**

The tool includes eight items, each of which is rated as being present (yes) or not present (no) and, for some items, not applicable or not reported. The items include (1) cohort, (2) control or comparison group, (3) pre-post intervention data, (4) random assignment of participants to the intervention, (5) random selection of participants for assessment, (6) follow-up rate of 80% or more, (7) comparison groups equivalent on sociodemographics, and (8) comparison groups equivalent at baseline on outcome measures. Together, items (1)–(3) summarize the study design, while the remaining items consider other common elements of study rigor. Inter-rater reliability was moderate to substantial for all items, ranging from 0.41 to 0.80 (median *κ* = 0.66). Agreement between raters on the total count of items endorsed was also substantial (*κ*_*w*_ = 0.66).

**Conclusions:**

Strengths of the tool include its applicability to a range of study designs, from randomized trials to various types of observational and quasi-experimental studies. It is relatively easy to use and interpret and can be applied to a range of review topics without adaptation, facilitating comparability across reviews. Limitations include the lack of potentially relevant items measured in other tools and potential threats to validity of some items. To date, the tool has been applied in over 30 reviews. We believe it is a practical option for assessing risk of bias in systematic reviews of interventions that include a range of study designs.

## Background

A 2010 article estimated that 75 trials and 11 systematic reviews are published in the medical field each day [1]; in 2016, this estimate was updated to 25 systematic reviews published each day [2]. Developing practical, effective tools to use in these reviews is critical to providing timely and useful summaries of a rapidly expanding and evolving evidence base.

Risk of bias in intervention studies has been defined as “the likelihood of inaccuracy in the estimate of causal effect in that study” [3]. In systematic reviews, assessing risk of bias of individual studies is essential in providing accurate assessments of the overall intervention effect. Many different tools have been developed to assess risk of bias. Several systematic reviews have identified an incredible array of tools (as many as 194 in one review) [4] that have been developed for different purposes, cover a range of study designs, and assess different domains of potential bias [4–6]. Given the diversity of purposes for which they were designed, each of these tools has unique strengths and weaknesses. However, the majority (87% according to one review) are specific to a single type of study design rather than encompassing a range of study designs, and few are validated [4]. While there have been a small number of assessments of the validity and reliability of existing tools in recent years [7–10], there is still generally limited information on which are best [3].

In this article, we present a tool for assessing risk of bias in both randomized and non-randomized intervention studies. The tool was developed by the Evidence Project, which conducts systematic reviews and meta-analyses of behavioral interventions for human immunodeficiency virus (HIV) in low- and middle-income countries. Specifically, we sought to develop a tool that would be appropriate for use across a range of study designs, from randomized trials to observational studies, and that would capture some of the main aspects of risk of bias among behavioral interventions in our field. Our goal here is to describe our risk of bias tool in sufficient detail that readers can interpret its use in Evidence Project reviews and apply it themselves in their own reviews if desired. We also evaluate reliability of the tool by assessing inter-rater agreement of both individual items and the total count of items.

## Methods

### The Evidence Project

The Evidence Project is a collaboration between researchers from the Medical University of South Carolina and the Johns Hopkins Bloomberg School of Public Health. Since 2002, we have conducted systematic reviews and meta-analyses of behavioral interventions related to HIV in low- and middle-income countries [11–26]. We have developed a database of articles included across all reviews, and from this database, we have conducted additional analyses, such as evaluating how condom use is measured across studies [27]. Given that our reviews include quasi-experimental studies, assessing the risk of bias of the design used is a critical need.

To be included in an Evidence Project review, studies must present quantitative comparisons of participants who received the interventions of interest compared with participants who did not. This can be accomplished through a study design that has either (1) pre-intervention/post-intervention comparisons of outcomes that compare people before and after the intervention is received or (2) multi-arm designs that compare people who received the intervention of interest with those selected for a control or comparison group. Comparison groups can include true controls who receive no intervention, comparison groups who receive a different kind of intervention, or comparison groups who receive a less-intensive version of the same intervention. Studies also have to be conducted in a low- or middle-income country, as defined by the World Bank [28].

All data extraction, including for the risk of bias tool, occurs in duplicate by trained masters or doctoral student research assistants (“coders”). Each study is assigned to two coders, who are instructed to individually complete a standardized coding form for the study. The two coders then come together to compare results and resolve any discrepancies through consensus or referral to a senior study team member if needed. All reviews follow PRISMA guidelines for reporting [29].

### Included study designs

In Evidence Project reviews, we include only studies that present a quantitative comparison between people who received the intervention of interest and people who did not. This could be a comparison of different people enrolled in two or more study arms (a multi-arm design) or it could be the same individuals measured before and after they received the intervention (a pre-post design). Studies that involve more than one study arm or group include randomized trials, non-randomized trials, case-control studies, cross-sectional studies, serial cross-sectional studies, and cohort studies. Studies that involve only one study group include before-after and time series designs.

When we use the term control group, we refer to study arms that do not receive any type of intervention. Comparison groups, on the other hand, receive an intervention but usually it is a different or less-intensive service than that provided to the intervention arm.

We determine the study design based on the analysis and results of a given paper. Sometimes the results reported in an article will be part of a larger trial, which may have a different study design than the design reported on in the paper. Of note, we do not use our tool to exclude studies from our reviews if they have high risk of bias; instead, we include all studies that meet our inclusion criteria, but use the risk of bias tool to consider which ones might present effect size estimates that are more likely to be closer to the actual effect. We then use this as we analyze and interpret the results, which can include selecting studies for meta-analysis.

### Development of the risk of bias tool

The Evidence Project risk of bias tool (also referred to in our publications as a rigor score) was developed with the goal of creating a simple but useful tool that would capture elements of study design and conduct that would facilitate comparison across the diverse range of study designs included in our reviews. Tools that provided separate criteria for randomized trials and non-randomized studies, while certainly relevant for the needs of each set of studies, failed to helpfully guide readers when both types of studies were included in a single review. The tool was developed through collaborative discussions between MS, KO, and JD and was informed by literature on research methods and validity in quasi-experimental designs, particularly Cook and Campbell’s classic book [30]. We have used the final tool in coding over 300 studies included in multiple published reviews [11–26]. We have thus had the chance to identify and refine a range of issues in its application.

### Inter-rater reliability

We calculated the inter-rater reliability for each tool item and for the total count of endorsed items using an illustrative set of 125 studies. Selected studies were included in seven previously published reviews conducted by the Evidence Project [13, 16–18, 21, 23, 26]. These reviews were selected for their range of included study designs. Ratings for each study were provided by two raters.

Cohen’s kappa (*κ*) [31] was estimated for individual bias tool items and weighted kappa (*κ*_*w*_) [32] for the count of endorsed items (the sum of individual item responses). All items are treated first as dichotomous. Since some of the items (4, 6, 7, 8) were rated using a categorical scale, we collapsed “not applicable” and “not reported” responses with “no,” reflecting a global assessment of whether the study did or did not get credit for having achieved that criterion. In addition, we also assessed agreement between raters for items (4, 6, 7, 8) when retaining Not Applicable and Not Reported as unique response options. Weighted kappa incorporates the magnitude of disagreement between raters on the count of endorsed items. IBM SPSS Statistics v24 was used to analyze data. We categorized agreement as poor (0.00), slight (0.01–0.20), fair (0.21–0.40), moderate (0.41–0.60), substantial (0.61–0.80), or almost perfect (0.81–1.00), in line with how other assessments of risk of bias tools have categorized agreement [33].

## Results

### Categories of assessment

The Evidence Project risk of bias tool includes eight items or criteria. For each item, if the study fulfills that criterion, a “yes” is put in that column of the risk of bias tool. If the study does not fulfill that criterion, a “no” is put in the column. Additional options for some items are “not applicable,” if the criterion does not apply given the study design, or “not reported,” if the fulfillment of the criterion cannot be determined by the information presented in the study. The eight items include (1) *cohort*, (2) *control or comparison group*, (3) *pre-post intervention data*, (4) *random assignment of participants to the intervention*, (5) *random selection of participants for assessment*, (6) *follow-up rate of 80% or more*, (7) *comparison groups equivalent on sociodemographics*, and (8) *comparison groups equivalent at baseline on outcome measures*. Table 1 presents these eight items with the response options for each. Table 2 presents an example of the completed rigor score for a selected review. As in this example, footnotes can be added to the table to clarify specific items or present results of sensitivity and sub-group analyses. Details of each item are listed below.

**Table 1:** Items, response choices, and inter-rater reliability estimates for the Evidence Project risk of bias tool

Risk of bias tool domains	Items	Response choices	Kappa (*κ*)^1^	
			Dichotomous	Categorical
Study design	1: Cohort	Yes, No	0.48	
	2: Control or comparison group	Yes, No	0.80	
	3: Pre/post intervention data	Yes, No	0.74	
Participant representativeness	4: Random assignment of participants to the intervention	Yes, No, NA	0.78	0.56
	5: Random selection of participants for assessment	Yes, No	0.41	
	6: Follow-up rate of 80% or more	Yes, No, NA, NR	0.67	0.55
Equivalence of comparison groups	7: Comparison groups equivalent on sociodemographics	Yes, No, NA, NR	0.65	0.56
	8: Comparison groups equivalent at baseline on outcome measures	Yes, No, NA, NR	0.59	0.50
Median kappa score (*κ*) across individual items 1–8			0.66	
Weighted kappa (*κ*_*w*_) of the total count of items (sum of individual item dichotomous responses)			0.66	

^1^Kappa estimates are reported for dichotomous (Yes, No) and categorical ratings when appropriate. Categorical response sets further classify binary No ratings as: No (reported), NA (not applicable), NR (not reported). Agreement was categorized as poor (0.00), slight (0.01–0.20), fair (0.21–0.40), moderate (0.41–0.60), substantial (0.61–0.80), or almost perfect (0.81–1.00)

**Table 2:** Example of a completed Evidence Project risk of bias tool from a review of interventions to increase HIV serostatus disclosure in low- and middle-income countries [16]

Study	Cohort	Control or comparison group	Pre/post intervention data	Random assignment of participants to the intervention	Random selection of participants for assessment	Follow-up rate of 80% or more	Comparison groups equivalent on sociodemographics	Comparison groups equivalent at baseline on disclosure
*Cognitive-behavioral support groups*								
Futterman et al.	Yes	Yes	Yes	No	No	No	No	Yes^1^
Jones et al.	Yes	Yes	Yes	Yes	No	Yes	No	No^1^
Kaaya et al.	Yes	Yes	Yes	Yes	No	No	Yes	NR
Mundell et al.	Yes	Yes	Yes	No	No	No	No	No
Sarnquist et al.	Yes	Yes	Yes	No	No	Yes	Yes	Yes
Snyder et al.	Yes	No	Yes	NA	No	No	NA	NA
Wouters et al.	Yes	Yes	No	No	Yes	Yes	NR	NR
*Home-based or peer/community health workers*								
MacNeil et al.	Yes	Yes	Yes	Yes	No	Yes	Yes	Yes
Ncama	No	Yes	No	No	Partial^2^	NA	No	NA
Rochat et al.	Yes	No	Yes	NA	No	Yes	NA	NA
Rochat et al.	Yes	No	Yes	NA	No	Yes	NA	NA
Wouters et al.	*See above*							
Zuyderduin et al.	Yes	Yes	Yes	No	No	Yes	Yes	NR^3^
*Partner notification*								
Brown et al.	No	Yes	No	Yes	No	NA	NR	Yes^4^
Henley et al.	No	Yes	No	No	No	NA	NR	NA

*NR* not reported, *NA* not applicable

^1^Calculated from additional data provided by authors

^2^Intervention group randomly selected, control group non-randomly selected

^3^Not calculable based on data provided in the article

^4^All participants were newly diagnosed, so presumably none had disclosed prior to the intervention

#### Cohort

Cohort analyses present data for a group of study participants followed over time. This may include pre-intervention to post-intervention analyses with or without a control or comparison group.

If the study includes a cohort that was followed over time and included multiple assessments with the same people, this criterion is met. If the study did not conduct multiple assessments with a cohort of individuals over time, this criterion is not met. For example, a study that used a serial cross-sectional design with different individuals (even if they are from the same population) completing the assessments would not be considering as having a cohort design.

#### Control or comparison group

Control or comparison groups are defined as analyses that compare those who received the intervention to those who did not. They may also include those who received a more-intensive versus less-intensive intervention. These include analyses that compare intervention, control and/or comparison groups, and cross-sectional analyses that are stratified by whether participants did or did not receive the intervention. This item does not include before-after analyses without stratification.

If the study included a control and/or comparison arm in addition to the intervention arm, this criterion is met. If the study only had an intervention arm, this criterion is not met.

#### Pre-post intervention data

Pre-post intervention outcome data is included in the risk of bias assessment, as it is common for studies to only assess outcome measures in the post-intervention catchments, especially for post hoc analyses and secondary study aims. Pre-post intervention data is present when the study presents outcome data for participants both before and after they receive the intervention. Such data may be presented at multiple time points either before or after the intervention.

If the study presents data from both before (baseline) and after the intervention, this criterion is met. If data are only presented post-intervention, this criterion is not met. If data are only presented pre-intervention, the study would not meet the inclusion criteria of having post-intervention evaluation data.

#### Random assignment of participants to the intervention

Random assignment to treatment groups assesses whether subjects were randomly assigned to treatment groups in multi-arm studies and includes group randomized designs. This criterion is nested within criterion for a control or comparison group in order to give added weight to designs which include randomization and control.

In multi-arm study designs, if participants are randomly assigned to the intervention and control/comparison arm, this criterion is met. This is true for both individual and group randomized designs. If participants self-select into the intervention or if assignment to the intervention is not random, this criterion is not met. If the study only has an intervention arm, this criterion should be listed as not applicable.

#### Random selection of participants for assessment

Random selection of subjects for assessments is assessed to consider whether there was a selection bias in study enrollment.

If authors use a probability sample to select participants (defined as a study in which the investigators pre-assess a sampling frame and randomly select groups or people from the specified population), this criterion is met. Similarly, if authors use a mixed sampling strategy but conducted random sampling for at least one part of that mixed strategy (for example, they have a non-probability sample of schools but then within schools randomly select students), then we consider this criterion as met because they randomly selected participants for assessment at some level, i.e., at one sampling frame. If authors used a non-probability sample (defined as a study in which the investigators use convenience or self-selected sampling strategies), then this criterion is not met.

#### Follow-up rate of 80% or more

Attrition of participants is measured at the final study follow-up. This is related to incomplete reporting, or loss-to-follow-up, that may introduce bias if participants who are retained are different than those who are not retained. One rule of thumb suggests that < 5% loss leads to little bias, while > 20% poses serious threats to validity [34]. This criterion is measured across the entire study population (all study arms).

If the entire study group had a follow-up rate of 80% or more, this criterion is met. If the follow-up rate was less than 80% at the final assessment, this criterion is not met. For studies that are post-intervention only or serial cross-sectional in nature, this criterion should be listed as not applicable.

#### Comparison groups equivalent on sociodemographics

Comparison group sociodemographic matching is assessed in multi-arm studies to determine if there are statistically significant differences in sociodemographic measures across arms at baseline. Sociodemographic measures may include characteristics such as age or gender, but should not include outcome measures. Study arms include intervention, control, or comparison groups.

If the study arms are equivalent on sociodemographic characteristics, this criterion is met. If there are significant differences between one or more of the study arms on socio-demographic characteristics, this criterion is not met. If the study only has one study arm, this criterion should be listed as not applicable. If the study has multiple study arms but the authors do not report whether the arms were equivalent on sociodemographic characteristics, this criterion should be listed as not reported.

#### Comparison groups equivalent at baseline on outcome measures

Comparison group outcome matching is assessed in multi-arm studies to establish whether there were statistically significant baseline differences in study outcome measures. As above, study arms include intervention, control, or comparison groups. Outcome measures are those which the intervention is trying to change; they generally include things like knowledge, attitudes, behaviors, or biological outcomes. There may be one or more outcome measures in any given study.

If the study arms are equivalent on outcome measures at baseline, this criterion is met. If there are statistically significant differences between one or more of the study arms on outcome measures at baseline, this criterion is not met. If the study only has one study arm, this criterion should be listed as not applicable. If the study has multiple study arms but the authors do not report whether the arms were equivalent on outcome measures at baseline, this criterion should be listed as not reported.

### Specific item considerations

Together, items (1) *cohort*, (2) *control or comparison group*, and (3) *pre-post intervention data* summarize the study design. Randomized controlled trials (RCTs) will meet all three criteria. Pre-post studies will meet criteria (1) and (3). Cross-sectional studies will meet only criteria (2), while serial cross-sectional studies that do not follow the same individuals will meet criteria (3) only. A study must meet at least one of these three criteria in order to be included in an Evidence Project review, according to our study design inclusion criteria.

The next three items focus on sampling and potential biases that may affect equivalence of the study groups or generalizability of the results. It is easy to confuse item (4) *random assignment of participants to the intervention* with item (5) *random selection of participants for assessment*. However, they are distinct in that (4) is related to randomization (internal validity), while (5) is related to selecting a representative sample (external validity). Importantly, studies may have one without the other. For example, in the HIV voluntary counseling and testing (VCT) efficacy study [35], individuals were recruited into the trial through advertisements about study services, with enrollment of people who responded to those advertisements (non-random selection of participants for assessment), but the enrolled individuals were then randomized to the intervention (VCT) and comparison (health information) study arms. Conversely, Magnani et al. [36] did the opposite: they used a probability sampling approach to recruit participants, but because they were evaluating a school-based sex education intervention that was not under their control, they relied on participant reports to assess who fell into the intervention and comparison groups. The item (6) *follow-up rate of 80% or more* is judged at the last follow-up in a given study, whether that is 1 week or 10 years after baseline. While this is based on a general rule of thumb and there is often at least some similarity in follow-up periods across studies within a given topic, it is common that studies have different follow-up periods, and so, this criterion assesses a different time period for different studies. One possible adaptation of the scale could be to select a common time period to assess for all studies included in a particular review and assess attrition at that time point.

Items (7) *comparison groups equivalent on sociodemographics* and (8) *comparison groups equivalent at baseline on outcome measures* consider potential confounding across study arms. For these two items, the risk of bias assessment relies on statistical significance, which is determined by sample size and may not reflect a clinically or programmatically meaningful difference. These measures also lump all sociodemographic measures and all outcome measures together; studies that measure more items are more likely to find at least one significant difference between groups by chance alone. Finally, others have noted that “baseline imbalances in observational studies that have no relationship with the outcome may not be consequential” [3]; instead, only those baseline variables which are highly correlated with the outcomes of interest may be relevant. This may be a concern with our tool, although we also believe authors generally include measures that are at least somewhat relevant to their topics.

There is some built-in dependency in the items in our tool, as the first three items assess study design and some of the later items are not relevant for all study designs. This is reflected in the not applicable response options. For example, items (7) and (8), which assess *comparison groups equivalent on sociodemographics* and *outcome measures*, are only relevant if the study design includes comparison groups, while item (6) *follow-up rate of 80% or more* is only applicable if there is a cohort.

### Summary across items

Sanderson has suggested three categories of quality assessments tools: scales, simple checklists, or checklists with a summary judgment. In early reviews conducted by the Evidence Project [11, 12, 15, 19, 20], our tool was the latter: we added up the number of criteria that had been met to create a final summary score for each study. This was helpful in allowing the reader to quickly assess quality across studies in the review, and we could use it in the text of reviews to easily keep in mind the general rigor when considering other aspects of the study, such as results. However, we came to realize that a summary score, while a convenient mental shortcut, may be misleading for several reasons. First, items in the tool are not independent; the lack of a cohort, for example, means that automatically, a follow-up rate will be not applicable and a pre-post study design by definition will not be able to randomize participants to the intervention. Second, the items may not be equally weighted. Therefore, while a score of 6 may appear to be twice as good as a score of 3, this may be inaccurate and potentially misleading. For these and other reasons outlined by others who have criticized summary scores [37, 38], we decided to stop reporting the overall summary score and instead leave the tool as a simple checklist; in more recent reviews [13, 14, 16–18, 21, 25, 26], we have presented the results of the items alone.

### Inter-rater reliability

Table 1 presents inter-rater reliability results. Inter-rater agreement was moderate to substantial for all items; kappa estimates ranged from 0.41 to 0.80 for each item. The median estimate across items was 0.66, indicating substantial agreement. As expected, kappa statistics were slightly lower when categorical response options were retained, but still always fell within the moderate agreement range. As an additional assessment of reliability, agreement between raters on the total count of items endorsed was substantial (*κ*_*w*_ = 0.66). All kappa estimates were significant at *p* < 0.001.

## Discussion

The Evidence Project tool assesses risk of bias in a range of different study designs with moderate to substantial reliability. This tool is one of many existing tools that systematic reviewers and others can select from. Viswanathan et al. [3] advocate that systematic reviewers should consider the following general principles when selecting a tool: (a) it should be specifically designed for use in systematic reviews, (b) be specific to the study designs being evaluated, (c) show transparency in how assessments are made, (d) address risk-of-bias categories through specifically related items, and (e) be based on theory or, ideally, empirical evidence. We believe our tool meets these criteria, though like any other tool, it has strengths and weaknesses and should be selected when it best meets the needs of a given review.

One strength of the Evidence Project risk of bias tool is its applicability to a range of study designs, from RCTs to case-control studies to cohorts to pre-post studies, and including both prospective and retrospective studies. Previous reviews have found that the majority (87%) of existing risk of bias tools are design-specific [4], although there may be clear benefits to including a range of study designs in a given systematic review [39]. This aspect also allows the tool to be used across a range of topics, thus facilitating comparison across topics; for example, we have found that some HIV prevention interventions (such as Condom Social Marketing [25]) rarely use RCTs, while other topics (such as school-based sex education [13]) are much more likely to do so. Our risk of bias tool highlights these differences when compared across reviews. Also facilitating comparability across reviews is the fact that the tool does not need to be adapted for each review, or for each included study. This distinguishes it from tools such as ROBINS-I [40], which asks reviewers to assess bias separately for each outcome included in each study (which may differ across studies and across review topics), or the Newcastle-Ottawa scale [41], which asks reviewers to select the most important factor for which studies should control (which may differ across review topics).

Other strengths of the Evidence Project risk of bias tool include its relative ease of use and clarity. The eight items are fairly straightforward and easy to assess, which should make data extraction less prone to error and easier for reviewers with less experience. The tool is also relatively easy for readers to interpret and read, as all information can be condensed into a single table with one row per study.

However, our tool also has some limitations. Some items, as noted above, may capture elements based on study features other than bias differentially across studies. For example, length of follow-up, which differs across studies, affects the 80% retention cutoff. Similarly, sample size and the choice of sociodemographic or outcome variables may both affect whether comparison groups are equivalent on these measures. While these items could be adapted for individual reviews, that would reduce the consistency across topics noted above.

Second, while our decision to change the tool to a simple checklist, rather than a checklist with a summary (numerical) judgment, avoids criticisms of summary scores, Viswanathan et al. have recently noted that this approach “devolves the burden of interpretation of a study’s risk of bias from the systematic reviewer to the reader.” [3] When we did present a summary score, readers found it easy to see differences in overall quality across included articles; without the summary score, we feel it has become more difficult to succinctly communicate overall risk of bias in presentation of the review results. An alternative may be to use individual items in the scale to create general categories, where studies could be ranked as “low,” “medium,” and “high” risk of bias. We have not done this to date, as the different items and domains do not assess an equal risk of bias; however, it could be considered by others using the tool.

Third, the Evidence Project risk of bias tool does not capture some elements of quality that other tools assess. For example, ROBINS-I [40] assesses bias in the classification of interventions, deviations from intended interventions, measurement of outcomes, and selection of the reported results. The Newcastle-Ottawa scale [41] considers items such as the case definition (for case-control studies) and ascertainment of exposure. The Cochrane Risk of Bias tool [42] includes items such as random sequence generation, allocation concealment, blinding of participants and personnel, blinding of outcome assessment, and selective reporting. For the Evidence Project, we focus on behavioral interventions that are often impossible to blind, and with few RCTs included in our reviews, items such as random sequence generation and allocation concealment are rare. In line with recommendations to “select the most important categories of bias for the outcome(s) and topic at hand” [3], we have found the categories in our risk of bias tool to be useful for an overall assessment of the diverse types of studies we see in the field of HIV behavioral interventions in low- and middle-income countries.

Inter-rater reliability was moderate to substantial for all items in our tool individually, and the median inter-rater reliability across items was substantial. This compares favorably to other risk of bias tools. Assessing the Cochrane Risk of Bias tool, Harding et al. found inter-rater agreement ranged from slight (*κ* = 0.13) to substantial (*κ* = 0.74) across items [33], while Armijo-Olivo et al. found inter-rater reliability was poor for both the overall score (*κ* =  0.02) and individual items (median *κ* = 0.19, range − 0.04 to 0.62). The Newcastle-Ottawa score has similarly been found to have fair inter-rater reliability overall (*κ* = 0.29), with individual items ranging from substantial (*κ* = 0.68) to poor (*κ* = − 0.06) [9]. The relative ease of use and clarity of items on our tool likely increased its reliability. However, as both reviewers were from the same study team, our inter-rater reliability results may have been more consistent than would be expected if the tool were applied by members of different groups. Several studies have found consistency may be even lower across different groups, such as Cochrane reviewers and blinded external reviewers [7] or across consensus assessments of reviewer pairs [8].

The Evidence Project risk of bias tool has been used in over 30 systematic reviews to date, including both Evidence Project publications [11–27] and other systematic reviews not connected with the Evidence Project [43–58]. Some of these reviews have changed the tools’ criteria slightly—for example, by using a 75% instead of 80% cutoff [44, 48, 49, 52, 54] or by adding an extra item for whether the study adjusted for confounding variables [44, 46, 48, 49, 52–54]. The Evidence Project risk of bias tool has been used in reviews of a range of topics, including in Cochrane reviews [14, 52] and reviews to inform World Health Organization guidelines [43–48, 50, 53]. We believe this widespread use in reputable settings, including by researchers outside our study team, provides at least some indication that others feel the tool is useful and has face validity.

## Conclusions

The Evidence Project risk of bias tool is a reliable tool for intervention studies that cover a range of designs, and it is relatively easy to apply. It is one option among many for consideration by systematic reviewers as they consider their specific review needs.

## Abbreviations

NA: Not applicable
NR: Not reported
RCT: Randomized controlled trial
